# Neurophysiological pitfalls in TTR-FAP Val30Met

**DOI:** 10.1186/1750-1172-10-S1-P31

**Published:** 2015-11-02

**Authors:** Isabel Conceição, Cristiana Silva, Mariana Costa, José Castro

**Affiliations:** 1 Centro Hospitalar Lisboa Norte - Hospital de Santa Maria, Department of Neurosciences, 1649-028, Lisbon, Portugal

## Introduction

The neurological hallmark of TTR-FAP is a length-dependent axonal neuropathy that initially involves the unmyelinated and small myelinated nerve fibers that mediate pain and temperature sensation, causing sensory disturbances that typically start in lower limbs. Subsequent degeneration of larger myelinated fibers results in large fiber sensory deficit and muscle weakness.

The disease can be difficult to recognize due to extreme phenotypic heterogeneity and nonspecific clinical symptoms even within the same mutation.

In TTR-FAP related to Val30Met mutation, different neuropathy phenotypes have been reported mainly in patients from non endemic areas as well in late onset cases.

## Case report

We described 3 TTR-FAP Val30Met early onset cases from endemic areas with a different neuropathy phenotype that can be easily misdiagnosed as a different entity. One case presented as a bilateral carpal tunnel syndrome without neuropathy; another with a demyelinating neuropathy with predominant upper limb involvement and other with a demyelinating neuropathy with conduction blocks mimicking a CIDP.

## Conclusion

TTR-FAP is frequently misdiagnosed, e.g. as idiopathic polyneuropathy or chronic inflammatory demyelinating polyneuropathy, and may be greatly under diagnosed. However, early accurate diagnosis of TTR-FAP is crucial for effective disease control.

